# Occurrence of Banned and Currently Used Herbicides, in Groundwater of Northern Greece: A Human Health Risk Assessment Approach

**DOI:** 10.3390/ijerph19148877

**Published:** 2022-07-21

**Authors:** Paraskevas Parlakidis, Maria Soledad Rodriguez, Georgios D. Gikas, Christos Alexoudis, Greivin Perez-Rojas, Marta Perez-Villanueva, Alejo Perez Carrera, Alicia Fernández-Cirelli, Zisis Vryzas

**Affiliations:** 1 Laboratory of Agricultural Pharmacology and Ecotoxicology, Department of Agricultural Development, Democritus University of Thrace, 68200 Orestias, Greece; pparlaki@agro.duth.gr (P.P.); solerodrigez@gmail.com (M.S.R.); calexoud@agro.duth.gr (C.A.); greperez@gmail.com (G.P.-R.); marta.perez@ucr.ac.cr (M.P.-V.); 2 Centro de Estudios Transdisciplinarios del Agua/CETA (UBA), Instituto de Investigaciones en Producción Animal/INPA (CONICET), Facultad de Ciencias Veterinarias, Universidad de Buenos Aires, Buenos Aires C1427CWO, Argentina; ceta@fvet.uba.ar (A.P.C.); inpa@fvet.uba.ar (A.F.-C.); 3 Laboratory of Ecological Engineering and Technology, Department of Environmental Engineering, School of Engineering, Democritus University of Thrace, 67100 Xanthi, Greece; ggkikas@env.duth.gr; 4 Centro de Investigación en Contaminación Ambiental (CICA), Universidad de Costa Rica, San Jose 2060, Costa Rica

**Keywords:** herbicides, metabolites, banned pesticides, groundwater, preferential flow, leaching

## Abstract

The presence of pesticide residues in groundwater, many years after their phase out in European Union verifies that the persistence in aquifer is much higher than in other environmental compartments. Currently used and banned pesticides were monitored in Northern Greece aquifers and a human health risk assessment was conducted. The target compounds were the herbicides metolachlor (MET), terbuthylazine (TER), atrazine (ATR) and its metabolites deisopropylatrazine (DIA), deethylatrazine (DEA) and hydroxyatrazine (HA). Eleven sampling sites were selected to have representatives of different types of wells. Pesticides were extracted by solid-phase extraction and analyzed by liquid chromatography. MET was detected in 100% of water samples followed by ATR (96.4%), DEA and HA (88.6%), DIA (78.2%) and TER (67.5%). ATR, DIA, DEA, HA, MET and TER mean concentrations detected were 0.18, 0.29, 0.14, 0.09, 0.16 and 0.15 μg/L, respectively. Obtained results were compared with historical data from previous monitoring studies and temporal trends were assessed. Preferential flow was the major factor facilitating pesticide leaching within the month of herbicide application. Moreover, apparent age of groundwater and the reduced pesticide dissipation rates on aquifers resulted of long-term detection of legacy pesticides. Although atrazine had been banned more than 18 years ago, it was detected frequently and their concentrations in some cases were over the maximum permissible limit. Furthermore, human health risk assessment of pesticides was calculated for two different age groups though drinking water consumption. In all examined wells, the sum of the HQ values were lower than the unity. As a result, the analyzed drinking water wells are considered safe according to the acute risk assessment process. However, the presence of atrazine residues causes concerns related with chronic toxicity, since ATR R values were greater than the parametric one of 1 × 10^−6^ advised by USEPA, for both age groups.

## 1. Introduction

Safe drinking water from surface- and ground-water is essential for human health, quality of life and socio-economic development of humanity and is a prerequisite factor for the human population [1]. The largest body of freshwater in the European Union (EU) is groundwater. In Greece, groundwater contributes to 13.9% of all renewable water resources. In Greece, the annual water consumption/requirements are mainly covered by groundwater use representing 36% in farming, 5% in public use and 1% in industrial production. Hence, the usual geophysical peculiarities of Greece render the groundwater pumping as the only source of drinking water [2].

Herbicides are generally considered the most economical and effective way to control weeds in agricultural and non-crop environments [3]. However, the increasing use of herbicides leads to natural waters contamination inducing public health and environmental threats. For instance, the toxicity effects of pesticides to non-target organisms, such as microbiota, fish, algae and aquatic invertebrates, were reported repeatedly by several authors [4,5]. Several factors can affect pesticides and their metabolites behavior in the environment. Physicochemical properties of pesticides such as ionization, water solubility, volatility, octanol-water partition coefficient, thermo-, photo- and hydrolysis stability combined with the soil properties including organic carbon content, texture, pH, clay mineral type, dissolved organic matter and cation exchange capacity have important roles on run-off, adsorption or leaching potential. Rainfall and irrigation intensity, biological processes (biodegradation) and the agricultural practices affect pesticide fate, too [6,7]. Point or nonpoint source pesticide pollution can cause groundwater contamination through leaching. Pesticides residues can reach groundwater in a short time (preferential flow) by macropores reducing the potential to be absorbed by soil or to be biodegraded [8]. Alternatively, pesticides may move through soil micropores slowly (matrix flow) and are available to interact with soil particles and microorganisms [9]. ATR, MET and TER have long DT_50_ values and low absorptivity in soil, which increase their potential to contaminate groundwaters [10,11].

Various directives regulated the presence of pesticides in groundwater such as Ground Water Directive [12], Drinking Water Directive [13], Water Framework Directive [14] and Directives about priority substances and environmental quality standards in the field of water policy [15]. The quality standards of drinking water, related to pesticides in EU, were set with maximum concentration of 0.1 μg/L and 0.5 μg/L of the presence of individual and total pesticides and metabolites, respectively [13]. In addition, EU has set environmental quality standards (EQS) for surface water bodies in the field of water policy for priority substances and certain other pollutants, including pesticides. According to this directive, the annual average EQS for atrazine was set to 0.6 μg/L and the maximum allowable EQS was set to 2 μg/L [15].

Recent studies showed that ATR is an endocrine disruptor causing disfunctions at normal human reproduction and development for both genders [16]. Furthermore, ATR was correlated to potential neurological and liver problems [5,17]. Regarding TER, humans are usually exposed to herbicide by inhalation and dermal contact, but the main exposure pathway is oral, through drinking water consumption [18]. Moreover, the TER and its metabolite desethyl-terbuthylazine were detected in hair samples of farm workers, but only slight to moderate irritation to the eyes and skin were observed [19]. MET belongs to Toxicity Category III for acute dermal, oral and inhalation effects, and to Toxicity Category IV for dermal and eye irritation [20]. Thorpe and Shirmohammadi [21] showed that the exposure of a mixture of herbicides containing MET to children caused a 7.6-fold increased chance of developing bone or brain cancer, leukemia and lymphoma compared to unexposed children. Hence, in Iowa and North Carolina, an increased risk of lung and prostate cancer was observed, when farmers were exposed to MET [22].

Northern Evros is one of the most important regions of agricultural economy in Greece. In addition, the extensive agricultural activity, the vicinity with transboundary rivers and different agricultural practices followed in Bulgaria and Turkey increase the complexity of studying the origin of pesticide pollution. Previous monitoring studies of Northern Evros showed medium detection frequency of ATR, MET, TER and ATR metabolites, e DIA, deethylatrazine (DEA) and HA [2,23]. Interestingly, despite the ban, ATR and its metabolites and MET have been frequently detected in high concentrations including water quality standards exceedances in numerous European countries such as Spain, Slovenia, Hungary, Portugal and Germany, over last decade [24,25,26,27,28]. High concentrations of TER were detected, too. For example, the maximum detected concertation in groundwaters in the Águeda River Basin from Spain and Portugal was 2.5 μg/L [24]. Surprisingly, a serious lack of human health risk assessment studies in groundwater is observed in Europe. Therefore, the aim of this study was to investigate the groundwater quality, the presence and the persistence of these compounds (i.e., ATR and its metabolites, MET and TER), and to characterize their temporal and spatial variability in the studied aquifer, 18 years after atrazine’s ban. Moreover, a chronic risk assessment of side effects on human health by consumption of contaminated drinking water was conducted. According to our knowledge, this study is the first attempt to correlate the human health risk assessment with the determination of pesticides residues in groundwater in Balkans and by extension in Greece.

## 2. Materials and Methods

### 2.1. Study Area Description

**Figure 1:**
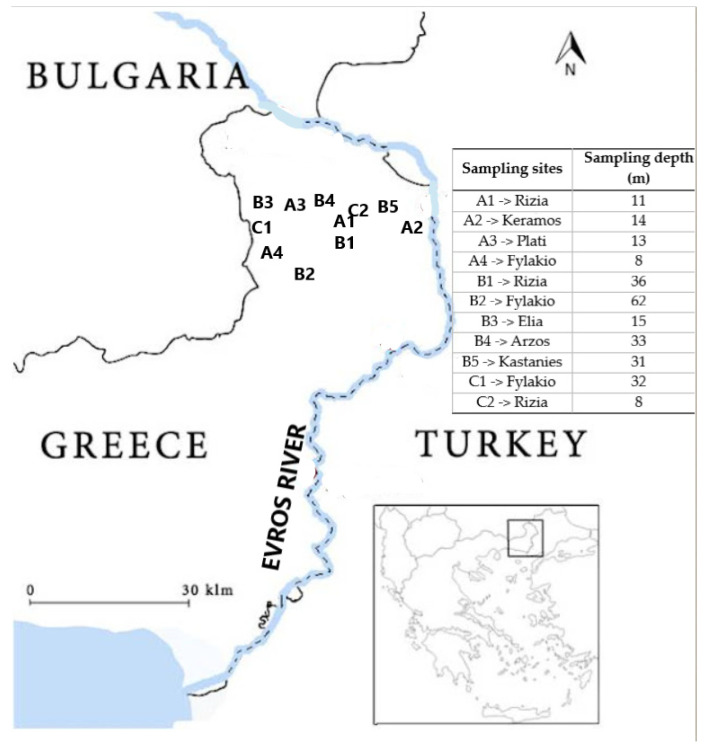
Study area of a transboundary aquifer (among Greece, Turkey and Bulgaria) and sampling sites. The capital letters A, B and C represent the experimental boreholes, drinking water wells and irrigation wells, respectively. Numbering identifies the villages close to sampling sites.

Sampling area was chosen taking into account the persistence of target pesticides in groundwater in Northern Evros, Thrace District, North Greece, as reported by previous studies and the extensive cultivation of wheat (2313.63 ha), cotton (2286.54 ha), sunflowers (1817.71 ha), maize (1695.18 ha) and beets (322.39 ha). Therefore, samples were taken from 11 sampling points, at Ardas Valley (third biggest arable area in Greece), divided in three Groups A sampling network of shallow groundwater was established during a previous study 20 years ago consisted of 4 experimental boreholes (Group A) [2]. Furthermore, 5 drinking water wells were included, which supply Orestiada town and local villages with potable water (Group B). In addition, two active irrigation wells were chosen (Group C) to include all available well types. Therefore, the sampling groups form was: Group A: A1, A2, A3 and A4, Group B: B1, B2, B3, B4 and B5 and Group C: C1 and C2. Sampling sites were located close to villages Rizia (A1, B1, C2), Keramos (A2), Plati (A3), Fylakio (A4, B2, C1), Elia (B3), Arzos (B4) and Kastanies (B5) (Figure 1).

Sampling was performed on a monthly basis for the period 28 March–5 September 2018, conducting 5 samplings. The samples (volume of 1 L) in triplicate were poured in dark polypropylene bottles. Then, they were transported to the Laboratory of Agricultural Pharmacology and Ecotoxicology, Department of Agricultural Development, at Democritus University of Thrace in ice-boxes and were stored in the dark at temperatures below −20 °C until analysis that was performed within a period no longer than 1 week. Experimental borehole samples were manually pumped using an experimental tube. The drinking water and irrigation wells were equipped with a pump system and samples were collected automatically before chlorination stage.

### 2.2. Chemicals and Instrumental Analysis

The pesticide standards had the highest available purity (>97%) and were purchased by Dr. Ehrestrofer GmbH (Augsburg, Germany). Physicochemical properties of studied compounds are shown in Table 1. The HPLC grades, acetonitrile, ethyl acetate, water and methanol for liquid chromatography were purchased by Riedel de Haen (Seelze, Germany). LiChrolut^®^ EN Polymer-based solid-phase extraction cartridges with 200 mg absorbent and 3 mL volume were purchased by Merck (Darmastdt, Germany).

Groundwater samples were prepared for instrumental analysis using solid phase extraction (SPE) for the multi-residue analysis as described by Papadakis and Papadopoulou-Mourkidou [30] with slight modifications. Water samples of 1 L were extracted by cartridges which were preconditioned with adding of 4 mL methanol followed by 4 mL deionized water. Samples were passed through cartridges at a flow rate of 5 mL/min. Target compounds were eluted with 7 mL methanol followed by 3 mL ethyl acetate. Next, samples were concentrated under nitrogen stream at 50 °C. Finally, samples were dissolved with 1.25 mL of methanol and stored at −20 °C until instrumental analysis.

Samples were analyzed by a HPLC/DAD equipped with autosampler (Finnigan Surveyor, Thermo Scientific, Boston, MA, USA). The analytical column C18 Speedcore 100 × 4.6 mm was purchased by Fortis Technologies Ltd. (Cheshire, UK). Chromatographic data were processed by the ChromQuest 5.0 software (Finnigan Surveyor, Thermo Scientific). The mobile phase was consisted of acetonitrile (A) and water (W). The flow was set at 1.0 mL/min and the gradient included the following steps: the elution began at 20-80/A-W, 20-80/A-W (0–20 min), 95-5/W-A (20–25 min), 95-5/A-W (25–26 min) and 20-80/A-W (26–33 min). Total run time was 40 min. The injection volume was 25 μL. The column oven temperature was adjusted at 30 °C. MET, TER, DEA and DIA were detected at 220 nm, while ATR and HA at 240 nm. For further confirmation of the target peaks, the UV absorption spectra taken at the apex of each sample were compared with those obtained from the standard solutions and control spiked samples. The quantification was performed using external working standard calibration curves (1, 10, 50 and 100 μg/mL). The accuracy (recovery) and precision (repeatability) of the analytical method were evaluated with the analysis of fortified (at 0.1 μg/g and 0.5 μg/g) tap water samples in duplicate. The limits of detection (LOD, μg/L) were determined as the lowest concentrations giving a response of three times the baseline noise of the analysis of three control samples. The limits of quantification (LOQ, μg/L) were determined as the lowest concentrations of a given compound in fortified samples that could be quantified with relative standard deviation lower than 20%. Positive detections of ATR, DIA, DEA, MET and TER were also confirmed with gas chromatographic analysis using a Trace 2000 gas chromatograph connected with the GCQ plus ion-trap mass spectrometer (Thermoquest, Austin, TX, USA). Gas chromatographic analysis was carried out on a 30 m × 0.25 mm I.D., 0.25 μm film thickness CP-SIL 8 CB (5% phenyl, 95% dimethylpolysiloxane) low bleed/MS column (Varian Analytical Instruments, Middelburg, The Netherlands) and the GC and MS operational conditions were those mentioned by Vryzas et al. [31]. Fortified tap water samples were used for the validation of the analytical method. Fortification levels includes the LODs, LOQs and the 0.1 μg/L. For all compound the LODs were ranged from 0.001 to 0.005 μg/L and LOQs from 0.01 to 0.05 μg/L. The recoveries were higher than 86% for all compounds with relative standard deviation lower than 15% at tested fortification levels.

### 2.3. Human Health Risk Assessment

Human health risk assessment was conducted for atrazine, metolachlor and terbuthylazine. According to Kim et al. [32] human health risk assessment of pesticides can provide information about the probability and the kind of effects to human population. In this case, oral exposure through drinking water consumption was considered as pathway to people. Risk assessment was divided to carcinogenic and non-carcinogenic and to two age groups, adults and children. Drinking water was provided to the local population by wells located close to the villages Elia, Arzos, Fylakio, Rizia and Kastanies (Figure 1).

### 2.4. Chronic Daily Intake (CDI)

CDI shows the estimated intake amount of pesticide per kilogram body weight (Equation (1)):

(1) $$\mathrm{CDIi}=\frac{D_{\mathrm{IP}}\times {\;\mathrm{EF}}_{i}\times {\;\mathrm{ED}}_{i}}{{\mathrm{BW}}_{i}\times \;\mathrm{AT}}$$

where D_IP_ is the average daily intake, EF_i_ is the exposure frequency (365 days per year for both age groups), ED_i_ is the exposure duration (6 and 70 years for children and adults, respectively), BW_i_ is equal to 70 kg for adults and 20 kg for children and AT is the average lifespan (2190 and 25,550 days for children and adults, respectively). The determination of the average daily intake (D_IP_) was estimated using the Equation (2), which was suggested by Muhammad et al. [33], Papadakis et al. [4] and Ali et al. [34]:

D_IP_= C_i_ × IR_i_ (2)

where C_i_ (μg/L) represents extreme and mean concentration of pesticide residues and IR_i_ shows the intake rate of water (0.87 L/day for children and 1.41 L/day for adults).

### 2.5. Hazard Quotient (Non-Carcinogenic Risk Assessment)

To calculate the hazard quotient (HQ), CDI was divided with the respective reference dose of each compound (Equation (3)):

HQ = CDI_i_/RfD (3)

where RfD is the acute toxicity reference dose [35].

The RfD values for atrazine, metolachlor and terbuthylazine, were 0.035, 0.015 and 0.008 (mg/kg-day), respectively [36,37]. When HQ values are equal or greater than 1, the exposed part of population is under health risk.

Multiple pesticides residues risk (HQs) can be calculated by the sum of HQ for individual pesticide using the Equation (4):

(4) $$\mathrm{HQs}=\sum_{i=1}^{n}\mathrm{HQi}$$

### 2.6. Carcinogenic Risk Assessment

Carcinogenic risk (R) was calculated by the Equation (5) [4,32]:

R = CDI × SF × ADAF(5)

where SF is the cancer slope factor (mg/kg-day), which reflects the possibility of the individual pesticide to cause cancer and ADAF is an age factor considering the early life pesticide exposure (3 for children and 1 for adults). Among the studied pesticides the only available SF value is for atrazine with value 0.22, provided by IRIS [36].

### 2.7. Statistical Analysis

An analysis of variance was conducted (ANOVA) for human health risk results using a mixed linear model according to Mas et al. [38]. The calculations were performed using the Excel Solver Add-in Package.

## 3. Results and Discussion

### 3.1. Concentrations and Detection Frequency

The sampling sites were in the Ardas valley, an aquifer vulnerable to pesticide contamination according to previous studies [5,23]. In general, the mean concentrations presented a wide range of values exceeding, occasionally, the maximum permissible limit of 0.1 μg/L in experimental boreholes (group A), drinking water wells (group B) and irrigation wells (group C) (Table 2).

According to the results shown in Table 3, the ATR mean concentration was above 0.1 μg/L in A2 and A4 sites. DIA mean concentration was found higher than 0.1 μg/L in A4, while HA surpassed it in A2. DEA mean concentration were higher than 0.1 μg/L in A1 and A4. TER presented limit exceedances in A2 and A4 and MET in all boreholes. As far as drinking water wells, ATR showed higher mean concentration than 0.1 μg/L in B1, B2 and B5. DIA mean concentration was higher in B1, B3 and B5. In HA case, mean concentration was above the limit in all drinking water wells. The mean concentration of MET was higher than 0.1 μg/L in B5, while TER limit exceedances were found in B1, B2 and B3. Concerning the irrigation wells, exceedances of 0.1 μg/L were observed for ATR, and DEA in both wells, for MET in C2 and for HA, DIA and TER in C1. Furthermore, the highest concentration of ATR, DIA, DEA, HA, MET and TER were 1.86, 2.91, 0.65, 0.46, 0.93 and 1.0 μg/L, respectively (Table 3).

Table 4 presents the percentage of positive samples in pesticide residues and the percentage of samples with concentration higher than permissible limits set by EU. Throughout the study, DIA and MET presented 90.9% detection frequency in water samples followed by ATR (87.3%), DEA and HA (81.8%) and TER (63.6%). The highest exceedance frequency of the maximum permissible limit in wells was observed in well B5 followed by C1, C2, B1 and B3. The measured values of pesticide concentrations in all sampling sites are presented by the box and whisker plot in Figure S1.

The maximum concentrations detected in this study are within the range of concentrations detected in groundwater samples at the European level, indicating that the established herbicide residues are an international issue. Menchen et al. [28], recorded the maximum concentrations for ATR (0.38 μg/L), ΜΕΤ (0.23 μg/L), DEA (0.12 μg/L), DIA (0.21 μg/L) and TER (0.90 μg/L). According to Meffe et al. [39], the maximum concentrations for TER in Italian groundwater was 29.05 μg/L. Considerable higher maximum concentrations were found in Spanish groundwater by Jurado et al. [40], for ATR (3.45 μg/L), MET (5.37 μg/L), DEA (1.98 μg/L) and TER (1.27 μg/L). Hernandez et al. [41] found that DIA was the most frequent detected compound (72%), followed by TER (50%), with maximum concentrations 1.42 μg/L for DEA, 0.40 μg/L for DIA and 0.46 μg/L for TER. Moreover, a third Spanish study in agricultural areas showed maximum concentration 0.33 μg/L for ATR, 0.37 μg/L for DEA, 0.34 μg/L for TER and 0.55 μg/L for MET with detection frequency ranged from 4% (DEA) to 68% (MET). The same study presented results from Portuguese groundwater in agricultural areas, too. TER had the highest concentration (1.88 μg/L) with frequency detection reached 56%, followed by ATR (0.19 μg/L) and detection frequency 25%. DEA and MET concentration were lower than 0.1 μg/L [24]. According to Korosa et al. [27], in groundwater samples from Slovenia ATR and DEA were detected at concentrations up to 0.228μg/L and 0.10 μg/L and their frequency of detection was 94.6% and 98.2%, respectively. In another study conducted in United Kingdom and France, the highest concentrations in British groundwaters for ATR, DIA, and DEA were 0.20, 0.10 and 0.16 μg/L, respectively, while the highest concentrations were lower than 0.1 μg/L, in France [42]. Table 4 presents the respective data of similar monitoring studies.

### 3.2. Historical Vulnerability of the Transboundary Aquifer to Contamination by Pesticide Residues

The fact that target compounds reached concentrations above the quality standard values for drinking water indicates that studies made during pesticides registration process are not always complying with the results from monitoring studies. It is estimated that less than 1% of the pesticides applied reach the target pest and the remaining distributed to various environmental compartments including groundwater bodies [43]. In a previous study was found that waters of that site constitute an aquatic mixture with different residence times and various leaching mechanisms are involved to the pollution of groundwater [44].

Similarly to the results obtained 15 tο 19 years ago extreme concentrations were observed in this study, indicating the presence of point source pollution sites nearby the studied wells. Target compounds had been monitored previously (between 1999–2003), at the same locations, before atrazine ban in EU [2]. Moreover, a similar study was conducted between 2010–2012 (data not shown), confirming the occurrence of ATR, DEA, DIA and MET (provided by the Greek Ministry of Agricultural Development). In order to have a better perspective on pollution temporal trends, the data of this study were compared with those of 1999–2003. Fifteen to nineteen years ago MET had been detected at least once in 63 % of the wells followed by ATR (61%), DEA (50%), alachlor (47%) and DIA (34%). According to Vryzas et al. [2], maximum concentrations for ATR (1.48 μg/L), DEA (0.76 μg/L), DIA (0.07 μg/L) and MET (1.54 μg/L) had been detected at the same drinking water wells sampled in this study and considerable higher pesticide concentrations were detected in shallow groundwater from experimental boreholes. This study confirms previously reported data on adsorption, degradation and leaching of selected pesticides [2,31,44,45,46]. Vulnerability of the aquifer to pollution depend on the land uses, soil properties, geological characteristics of the unsaturated zone, the hydraulic properties, the depth of the vadose zone and the leaching potential or physicochemical properties of the contaminant.

According to previous studies focused on this area, ATR degraded faster than MET in all soils of the vadoze zone and the biotransformation rates of both compounds decreased as the soil depth increased. Hence, the chronic presence of ATR in field is indicated by the higher biotransformation rate of ATR in soil taken from the middle of a studied field in comparison with soil sampled from the field margins [2]. The major metabolites of ATR and MET were found at higher concentrations in the 10–20 cm layers of all soil cores studied 0–110 cm below ground surface. However, the enhanced biodegradation rate of ATR in these soils is not enough to prevent the contamination of groundwater bodies. Our results have been observed by other studies, too. According to McMahon et al. [47], Kolpin et al. [48] and Steele et al. [49] degradation rates of triazine parent compounds are slower than their transport rates in groundwater. Moreover, Vonberg et al. [25] and Jablonowski et al. [50] conducted a solid samples analysis contaminated with atrazine from a lysimeter and agricultural field, respectively, reporting the persistence of the parent compound for over 20 years after its last application, followed by possible leaching. 

Adsorption studies of ATR, DEA, DIA, HA and MET were also conducted in soils from five different depths (i.e., 0–10, 10–20, 20–40, 40–80 and 90–110 cm below ground surface). The present study revealed that when pseudo-equilibrium stage reached, the amount of the adsorbed compounds accounted only for 10, 14, 27, 43 and 94% of the initial amount of DEA, DIA, ATR, MET and HA, respectively, spiked to the soils. According to this study, it was expected that more than 57 and 73% of the applied dose of MET and ATR, respectively, to be desorbed into the soil water and be available for leaching to deeper soil layers [45].

In addition, the low adsorption capacity of ATR and MET within soil profile of the studied area, proved that the preferential flow is a major pesticide leaching mechanism in this area since pollutants can reach the saturated zone of the aquifer through preferential flow paths (shrinkage of the clay minerals, plant roots and earthworms forming burrows) without going through chromatographic flow within the unsaturated zone and thereby circumventing the degradation processes [46]. Studies on the apparent age of the studied aquifers shown that the residence time of groundwater bodies ranged from 1.2 to 50 years [2].

The leaching mechanisms prevailed in this area has been also studied in an extensive four-year field experiment focused on soil water samples taken from 0–25, 35, 60, 100 and 160 cm below ground surface [46]. According to this study, MET, ATR, DEA and DIA were detected in more than 67% of the total soil water samples. The main conclusion of this study was that the corn-applied herbicides have been leached below the surface soil via macropore-dominated pathways in less than one month after their application. Agricultural practices (application of pesticides and sprinkler irrigation) used in this area, soil structure and hydrogeological conditions increase the leaching potential of pesticides in the studied area. It is worth notice that alachlor, another banned herbicide, with very limited half-life period (DT_50field_ = 14 days) had been detected in soil water of the studied area at concentrations greater than 0.1 mg/L up to 40 months after its application.

Irrigation is usually carried out by self-propelled sprinkler irrigation systems or basin irrigation systems multiple times in summer period. These types of irrigation system provide high volumes of high-pressure water, which, in combination with rainfall, can exacerbate the phenomenon of leaching [46]. Moreover, Nouma et al. [51] mentioned that basin irrigation systems can favor pesticides leaching to groundwaters, too. As recommend by Vryzas, et al. [46], the late pesticide application, use of drip instead of sprinkler irrigation and delayed first irrigation seem to be the major management actions according to good agricultural practice that prevent pesticide leaching to groundwater in a semiarid Mediterranean region. The limited spatial and temporal variation of concentration levels observed in studied wells indicates a continuous load of the aquifer with the target compounds. The continuous use of high amounts of atrazine for more than 30 years was enough to contaminate the soil and aquifer and to be detected with its metabolites in groundwater 15 years after its last use (2004). However, illegal applications cannot be excluded since the studied area is 20 km from Turkish and Bulgarian borders and illegal trade of banned pesticides had been observed.

**Figure 2:**
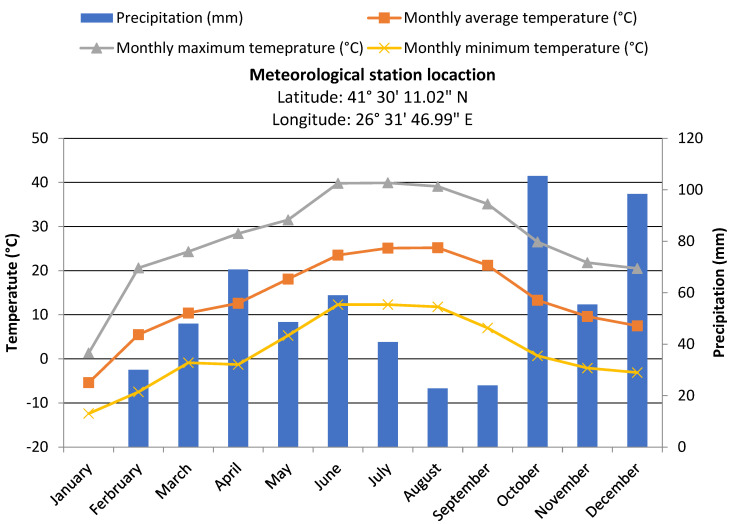
Meteorological data during the sampling period.

The variability and seasonality of temperature and rainfall impact significantly on the fate of pesticides within soil zone. Along the study, the temperatures were ranged between 5.3 and 39.9 °C, reaching extremely high values. The vapor pressures of pesticides can be increased as the temperature increases. In this study, pesticides have low vapor pressures (Table 2) and therefore volatilization could not cause losses, except for DEA. Moreover, high rainfall values were observed in May and June, 48.6 and 59 mm, respectively (Figure 2). Heavy rainfall could induce the remobilization of organic pollutants adsorbed in soil or sediments, especially lipophilic compounds such as ATR, TER and MET. Moreover, high rainfall intensities can cause overflow events and increase the frequency of leaching phenomenon, which can also be more rapid under rainy conditions [52,53].

The MET and ATR effectiveness against corn weeds and the limited available herbicides resulted their extensive use during the period of 1980–2005. ATR withdrawal brings out the TER as the most used herbicide, until nowadays. Application rates are similar, ranged from 600 to 1200, 900 to 1800 and 860 to 1200 gr/ha for ATR, MET and TER, respectively. The cropping system and major crops has been gradually changed from 2005 till now. However, field crops are still the major crops in the area and the irrigation practices are the same that were used 20–40 years ago. TER applied by farmers in agricultural area of Ardas valley as a pre-emergence herbicide for maize, cotton and beet cultivations in April, replacing the banned ATR. The extended treatment period of TER coincides with the significant rainfall period in the studied area. The use of a basic measure also identifies TER as a moderate leachers: in fact, TER has a GUS (groundwater ubiquity score) score of 2.19, indicating that it is a leacher [54].

In laboratory investigations with various top-soils, the degradation of [^14^C] TER was examined to determine which soil features would enhance metabolite production. After a 45-day incubation period, the concentration of TER dropped by 30%, while desethylterbuthylazine was generated at the same time. In different soils, the TER half-life ranged from 88 to 116 days. In addition, a research team hypothesized that TER is more readily absorbed by soil than atrazine, which could shield the molecule against biodegradation [55].

Commercial plant protection products bentazone, terbuthylazine and metolachlor were used to investigate the degradation and leaching of bentazone, TER and S-metolachlor, as well as their metabolites applied to a weighable, monolithic, high precision lysimeter with a loamy, sandy soil. Bentazone decomposed faster in soil than TER and S-metolachlor, but TER metabolization after the second application resulted in higher formation of desethylterbuthylazine and TER hydroxylation. On day 12, the greatest concentration of TER (310 g/kg) was found while the topsoil contained 16 g/kg of terbuthylazine on day 150, indicating leaching losses [56].

### 3.3. DEA to ATR Ratio

The DEA to ATR ratio (DAR) has been used to categorize point and non-point source pollution of groundwater and, in order to, characterize the degradation and transport of ATR in response to its metabolite DEA. This ratio can give us an indication of the major leaching mechanisms contribute to the pollution of groundwater and the capacity of the unsaturated zone to biodegrade ATR to DEA. During the transport of atrazine through chromatographic flow within the biological more active unsaturated zone it could be metabolized in significant amounts by microorganisms to DEA [2,57,58]. In such cases the DAR would have values higher than 0.4, or even close to 1.0. In contrary, when atrazine bypasses the vadose and enters the saturated zone through preferential flow the contact time between atrazine and soil microbial community could be shorter and, therefore, the DAR ratio would be less than 0.4. The DAR ratio can provide information about atrazine leaching behavior based on the fact that atrazine represents a closer adsorption capacity to DEA than to HA in spite of HA was found as the main metabolite of atrazine at same area. In addition, this soil can adsorb higher amount of atrazine than DEA. Therefore, DEA can be leached faster than atrazine through chromatographic or preferential flow [46]. The calculated DAR in this study was found to be higher than one in some cases, and lower than one in most samples (Table 5), indicating that contamination in some cases comes from diffuse sources but most probably the bound atrazine was gradually desorbed from the soil matrix to the soil water and moved to groundwater through preferential flow [25,46,59]. Although DAR values can be used to characterize the predominant leaching mechanisms in the studied aquifer, the different types of wells (experimental boreholes, drinking water wells and irrigation wells) used in this study and the respective differences in construction techniques restrict the estimation of the various mechanisms involved in the leaching processes.

Our results are in agreement with those of Vryzas et al. [44], conducted in the same area 15–19 years ago, who found similar DAR values few months after the application of atrazine. Overall, atrazine’s degradation products showed higher concentrations than did the parent compound. DIA exhibits a large range of concentrations varying between 0.01 μg/L and 2.91 μg/L. According to biotransformation studies conducted in the soil profile of the studied area HA was the most frequently detected metabolite and with the highest concentrations. The second most frequently detected degradation product in soil was DEA, followed by rare DIA detections [2]. The overwhelming majority of soil water samples with DEA presence, showed DEA had greater concentrations than DIA and the ratio values CDEA/CDIA reached 33 [51]. DEA (50% of groundwater samples) was more frequently detected than DIA (34% of groundwater samples) in an extensive groundwater monitoring program conducted in the same area 15–19 years ago [2]. Contrary to previous reported data, in this study, atrazine and its metabolites were detected with equal frequency of detection.

### 3.4. Human Health Risk Assessment

An extended discussion was preceded related to the presence, occurrence and distribution reasons of target pesticides at studied area. Results on human health risk assessment are presented in Table 6. Although, the HQ values for individual pesticide did not exceed the value 1, the estimated non-carcinogenic risk for children was higher, when compared to adults. The HQ values for mean pesticides concertation were ranged between 0.0171 to 0.1913 for adults and between 0.0393 to 0.5752 for children. The highest mean values are reported to metolachlor and the lowest to atrazine. The highest HQ values were determined in drinking water well close to Rizia namely, 0.2507 and 0.7817 for adults and children, respectively. Similar HQ values for atrazine and metolachlor in surface water were reported by Papadakis et al. [4], in a Greek study. The risk level for TER was low with HQ values lower than 0.6.

The sum of HQ values did not reach the unity in all studied wells. The greatest cumulative potential risk was determined in the Rizia well with values 0.2836 and 0.8299 for adults and children, respectively. The lowest potential risk has the Elia well, with values lower than 0.4. Consequently, according to the acute risk assessment, the studied drinking water wells are characterized as safe.

Oppositely, the carcinogenic risk assessment showed high values. In all cases, ATR R values were higher than the parametric one of 1 × 10^−6^ recommended by USEPA, for both age groups, showing that the local population were under carcinogenic risk (Table 6). The water consumption through the Fylakio well presents the highest risk, while the Arzos well was the lowest. The R values are ranged between 0.0002–0.0018 for adults and 0.0012–0.0181 for children. Previous study conducted in Greece, indicated high carcinogenic risk only for children [4].

## 4. Conclusions

All pesticides were detected in both shallow and deep ground-water bodies (experimental boreholes, drinking or irrigation water wells). DIA and MET presented the highest detection frequency in water samples, while TER and DIA showed the highest exceedance frequency of the EU permissible limit. Perennially, these herbicides were used in a variety weed-sensitive cultivations, including wheat, beet, cotton, maize and sunflower. The type of crops grown in a region largely determines the types of pesticides that were used, which in turn affects the residues of pesticides that are typically found in the groundwaters of studied region. Moreover, illegal used of banned pesticides cannot be excluded from a transboundary area. Despite agricultural use of atrazine has been banned in Greece for more than 18 years ago, atrazine and its metabolites residues are still detected in groundwater of the region, indicating their high persistence in saturated zone. The repeated application of studied pesticides could lead to enhanced biodegradation, as previously reported in the studied area, but the remaining amounts of bound residues was gradually desorbed from the soil matrix to the soil water and moved to groundwater through preferential or chromatographic flow. Furthermore, the detection of extreme concentrations suggests the presence of occasional point-sources pollution. However diffuse sources cannot be excluded. These observations were confirmed by DAR ratios and the frequency of detection of parent compounds and their respective metabolites, suggesting multi-mixed leaching. The drinking water consumption for local people is safe considering the acute risk assessment. However, the atrazine R values suggested high carcinogenic risk. We suppose that EU regulatory agencies could benefit from the linking of monitoring data with probabilistic human models in order to propose efficient pollution management methods.

## Figures and Tables

**Table 1:** Physicochemical properties of target compounds [29].

Compound	Soil Degradation DT_50_ (Field)	Dissociation Constant (pKa) at 25 °C	Water Solubility at 20 °C (mg/L)	Octanol-Water Partition Coefficient at 20 °C (LogK_ow_)	Vapour Pressure at 20 °C (mPa)	GUS Leaching Potential Index
ATR	29	1.7	35	2.7	0.039	2.57
DIA	-	-	980	1.15	-	-
DEA	45	-	2700	1.5	12.44	3.24
HA	-	-	5.9	2.09	1.131	
MET	21	-	530	3.4	1.7	2.36
TER	21.8	1.9	6.6	3.4	0.152	2.19

ATP: atrazine, DIA: deisopropylatrazine, DEA: deethylatrazine, HA: hydroxyatrazine, MET: metolachlor and TER: terbuthylazine.

**Table 2:** Statistics of pesticides concentrations (μg/L).

	Parameter	Sampling Sites										
		A1	A2	A3	A4	B1	B2	B3	B4	B5	C1	C2
ATR	Mean	0.02	0.23	0.10	0.28	0.23	0.45	0.03	0.05	0.30	0.20	0.14
	Median	<0.00	0.01	0.01	0.01	0.11	0.18	0.02	0.01	0.24	0.16	0.17
	Max	0.07	0.50	0.30	0.55	0.63	1.86	0.06	0.05	0.49	0.56	0.26
	*n*	5	5	5	5	5	5	5	5	5	5	5
DIA	Mean	0.06	0.05	0.06	0.14	0.14	0.09	0.26	0.08	0.22	1.99	0.06
	Median	0.02	<0.00	0.02	<0.00	0.15	0.09	0.20	0.02	0.22	2.01	0.04
	Max	0.14	0.14	0.12	0.45	0.12	0.13	0.49	0.15	0.31	2.91	0.14
	*n*	5	5	5	5	5	5	5	5	5	5	5
DEA	Mean	0.13	0.05	0.05	0.18	0.13	0.05	0.17	0.05	0.28	0.23	0.20
	Median	0.01	0.01	<0.00	<0.00	0.09	0.01	0.07	0.03	0.32	0.32	0.20
	Max	0.46	0.05	0.05	0.65	0.27	0.05	0.45	0.06	0.13	0.42	0.55
	*n*	5	5	5	5	5	5	5	5	5	5	5
HA	Mean	0.07	0.30	0.05	0.07	0.05	0.05	0.08	0.05	0.06	0.13	0.10
	Median	0.05	0.01	0.02	0.00	0.01	0.02	0.05	0.01	0.06	0.06	0.05
	Max	0.20	0.08	0.07	0.27	0.32	0.08	0.16	0.05	0.46	0.30	0.29
	*n*	5	5	5	5	5	5	5	5	5	5	5
MET	Mean	0.18	0.60	0.11	0.24	0.06	0.05	0.06	0.05	0.22	0.10	0.14
	Median	<0.00	<0.00	0.04	<0.00	0.05	0.01	0.03	<0.00	0.13	0.11	0.12
	Max	0.58	0.23	0.20	0.93	0.12	0.04	0.15	0.05	0.52	0.17	0.41
	*n*	5	5	5	5	5	5	5	5	5	5	5
TER	Mean	0.02	0.45	0.05	0.15	0.25	0.14	0.05	0.04	0.16	0.20	0.09
	Median	0.01	0.80	<0.00	0.10	0.21	0.16	0.02	<0.00	0.17	0.11	0.13
	Max	0.06	1.00	0.20	0.34	0.16	0.36	0.07	0.11	0.16	0.56	0.16
	*n*	5	5	5	5	5	5	5	5	5	5	5

*n* = number of samples in triplicate.

**Table 3:** Summary of positive samples in pesticide residues and exceedances of the EU permissible limit.

Sampling Sites		Positive Samples (%) **						Samples with Concentration Higher than 0.1 μg/L (%)					
	N *	ATR	DIA	DEA	HA	MET	TER	ATR	DIA	DEA	HA	MET	TER
A1	5	80	80	60	60	80	60	0	40	20	20	40	0
A2	5	80	80	80	80	80	40	0	20	0	0	20	40
A3	5	80	80	40	80	80	20	40	20	0	0	40	20
A4	5	80	80	40	80	80	60	40	40	20	20	20	60
BI	5	80	100	100	80	100	100	60	60	40	0	40	100
B2	5	80	100	100	80	100	60	60	40	0	0	0	60
B3	5	100	100	100	100	100	80	0	100	40	40	20	0
B4	5	80	80	80	80	80	40	0	40	0	0	0	20
B5	5	100	100	100	100	100	80	100	100	80	40	60	80
C1	5	100	100	100	60	100	80	60	100	60	40	60	80
C2	5	100	100	100	100	100	80	60	40	60	40	60	60
Total	55	87.3	90.9	81.8	81.8	90.9	63.6	38.2	54.5	29.1	18.2	32.7	47.3

* Number of samples in triplicate. ** Positive samples in pesticide residues are considered when pesticide concentrations are ≥ of LOQs.

**Table 4:** Pesticides concentration from different European countries.

Reference	Maximum Concertation (μg/L)	Detection Frequency (%)	Samples > 0.1 µg/L (%)	Year	Country
Menchen et al. [28]	ATR (0.38) MET (0.23) TER (0.90) DIA (0.21) DEA (0.12)	ATR (4.45)	ATR (1.91)	2017	Spain
		MET (1.91)	MET (0.63)		
		TER (12.1)	TER (2.81)		
		DIA (8.28)	DIA (0.32)		
		DEA (5.73)	DEA (0.32)		
Meffe et al. [39]	TER (29.05)	not specified	not specified	2014	Italy
Jurado et al. [40]	ATR (3.45) MET (5.37) TER (1.27) DEA (1.98)	not specified	not specified	2012	Spain
Hernández et al. [41]	TER (1.42) DIA (0.46) DEA DEA (0.40)	TER (50) DIA (72) DEA (35)	TER (15) DIA (35) DEA (8)	2008	Spain
Sanchez-Gonzalez et al. [24]	ATR (0.37) MET (0.55) TER (0.34) DEA (0.34)	ATR (4) MET (100) TER (70) DEA (8)	ATR (4) MET (48) TER (36) DEA (4)	2013	Spain
Sanchez-Gonzalez et al. [24]	ATR (0.19) MET (0.05) TER (1.89) DEA (0.08)	ATR (30) MET (25) TER (55) DEA (26)	ATR (5) MET (0) TER (25) DEA (0)	2013	Portugal
Korosa et al. [27]	ATR (0.23)	ATR (94.6)	not specified	2016	Slovenia
	MET (0.068)	MET (38.8)			
	TER (0.03)	TER (574)			
	DIA (0.02)	DIA (17.9)			
	DEA (0.1)	DEA (98.2)			
Lapworth et al. [42]	ATR (0.2) DIA (0.1) DEA (0.16)	ATR (12.5) DIA (9.6) DEA (20)	not specified	2015	England
Lapworth et al. [42]	ATR (0.69) DIA (0.47) DEA (0.13)	ATR (73) DIA (60) DEA (47)	not specified	2015	France

**Table 5:** Percentage (%) of samples with DAR value higher than 1.

Sampling Site	Percentage (%)
A1	50
A2	25
A3	50
A4	50
B1	0
B2	0
B3	80
B4	50
B5	0
C1	40
C2	25

**Table 6:** HQ and R indexes of pesticides through drinking water consumption, pumped by drinking wells.

		Rizia (B1)		Fylakio (B2)		Elia (B3)		Arzos (B4)		Kastanies (B5)	
	Index	Adult	Child	Adult	Child	Adult	Child	Adult	Child	Adult	Child
ATR	HQ_m_^a^	0.0422	0.1262	0.0684	0.2162	0.0171	0.0393	0.0184	0.0416	0.0515	0.1563
	HQ_h_^b^	0.0895	0.2916	0.2305	0.7839	0.0225	0.0548	0.0205	0.0509	0.0737	0.2333
	R_m_^c^	0.0003	0.0029	0.0005	0.0050	0.0001	0.0009	0.0001	0.0010	0.0004	0.0036
	R_h_^d^	0.0007	0.0067	0.0018	0.0181	0.0002	0.0013	0.0002	0.0012	0.0006	0.0054
MET	HQ_m_	0.1913	0.5752	0.1342	0.3757	0.0751	0.1692	0.0821	0.1941	0.1432	0.4063
	HQ_h_	0.2507	0.7817	0.2502	0.7818	0.0985	0.2505	0.1206	0.3254	0.2238	0.6881
TER	HQ_m_	0.0501	0.1235	0.0391	0.0787	0.0501	0.1233	0.0402	0.0901	0.0512	0.1303
	HQ_h_	0.0672	0.1833	0.0459	0.1070	0.0758	0.2136	0.0481	0.1172	0.1619	0.5132
HQ SUM	HQi	0.2836	0.8299	0.3058	0.6779	0.1403	0.3318	0.1407	0.3258	0.2459	0.6929

^a^ HQ_m_ hazard quotient (mean concentration), ^b^ HQ_h_ hazard quotient (highest concentration), ^c^ R_m_ carcinogenic risk (mean concentration) and ^d^ R_h_ carcinogenic risk (highest concentration).

## Data Availability

The datasets used and/or analyzed during the current study are available from the corresponding author on reasonable request.

## Supplementary Materials

The following supporting information can be downloaded at: https://www.mdpi.com/article/10.3390/ijerph19148877/s1, Figure S1: Box-whisker plots of concentrations of pesticides in sampling points: (a) atrazine, (b) DIA, (c) DEA, (d) HA, (e) terbuthylazine and (f) metolachlor. The caps at the end of each box indicate the extreme values (minimum and maximum), the box is defined by the lower and upper quartiles, and the line inside the box denotes the median value. The pesticide concentrations below the LOQs are considered as zero.
