# Supplementary material for: Occurrence of Banned and Currently Used Herbicides, in Groundwater of Northern Greece: A Human Health Risk Assessment Approach
Source: Int J Environ Res Public Health. 2022 Jul 21;19(14):8877. doi: 10.3390/ijerph19148877 (PMC9323306; doi:10.3390/ijerph19148877)
Supplement: Supplementary file 1 [file ijerph-19-08877-s001.zip › ijerph-1780715-supplementary.pdf]

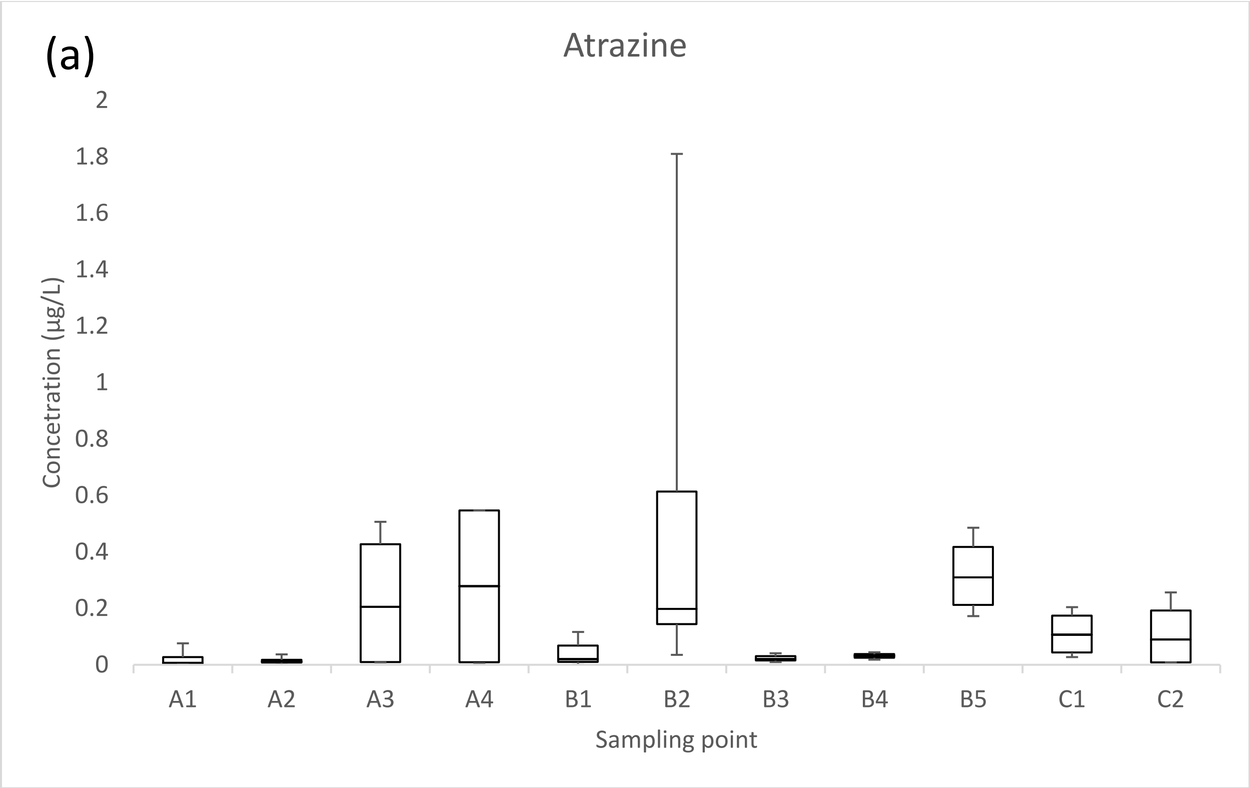

(a)
Atrazine
Concetration (µg/L)
2
1.8
1.6
1.4
1.2
1
0.8
0.6
0.4
0.2
0
A1
A2
A3
A4
B1
B2
B3
B4
B5
C1
C2
Sampling point

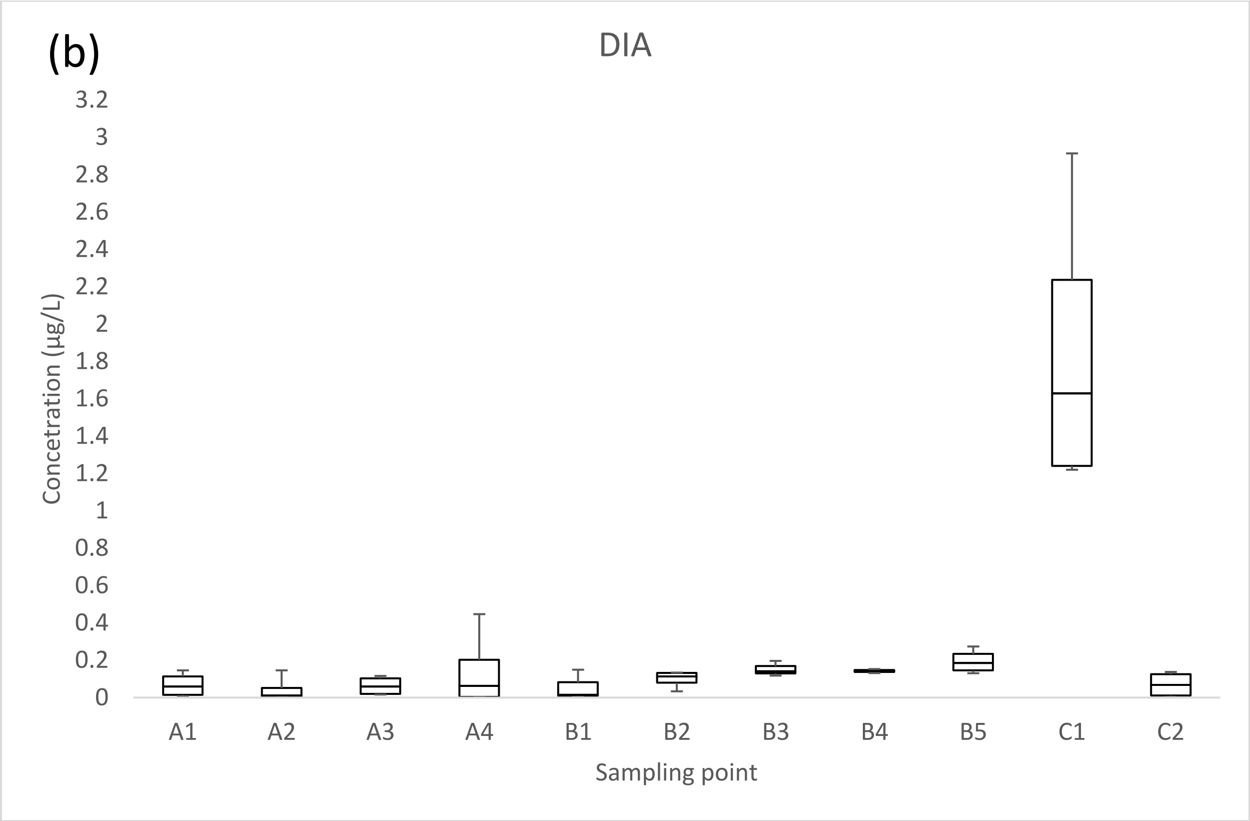

(b)
DIA
Concetration (µg/L)
3.2
3
2.8
2.6
2.4
2.2
2
1.8
1.6
1.4
1.2
1
0.8
0.6
0.4
0.2
0
A1
A2
A3
A4
B1
B2
B3
B4
B5
C1
C2
Sampling point

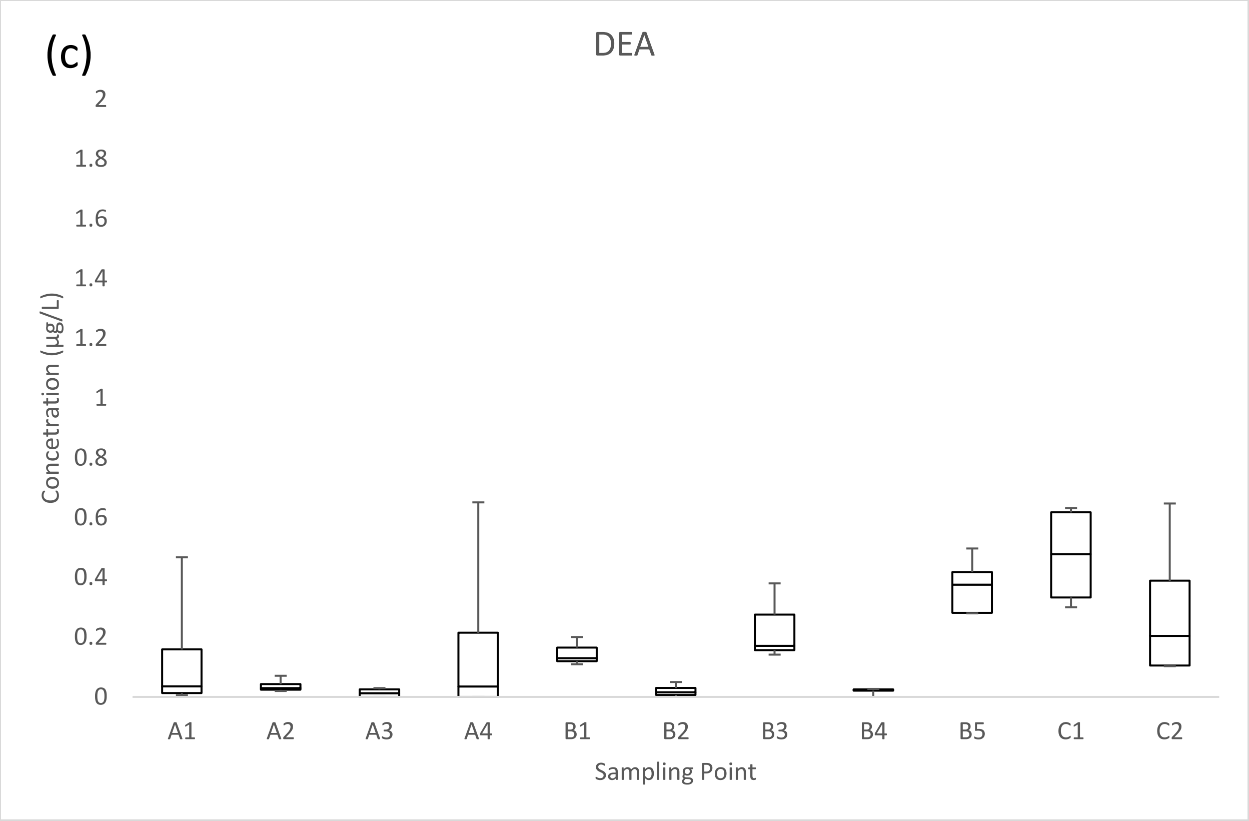
(c)
DEA
Concetration (µg/L)
2
1.8
1.6
1.4
1.2
1
0.8
0.6
0.4
0.2
0
A1
A2
A3
A4
B1
B2
B3
B4
B5
C1
C2
Sampling Point

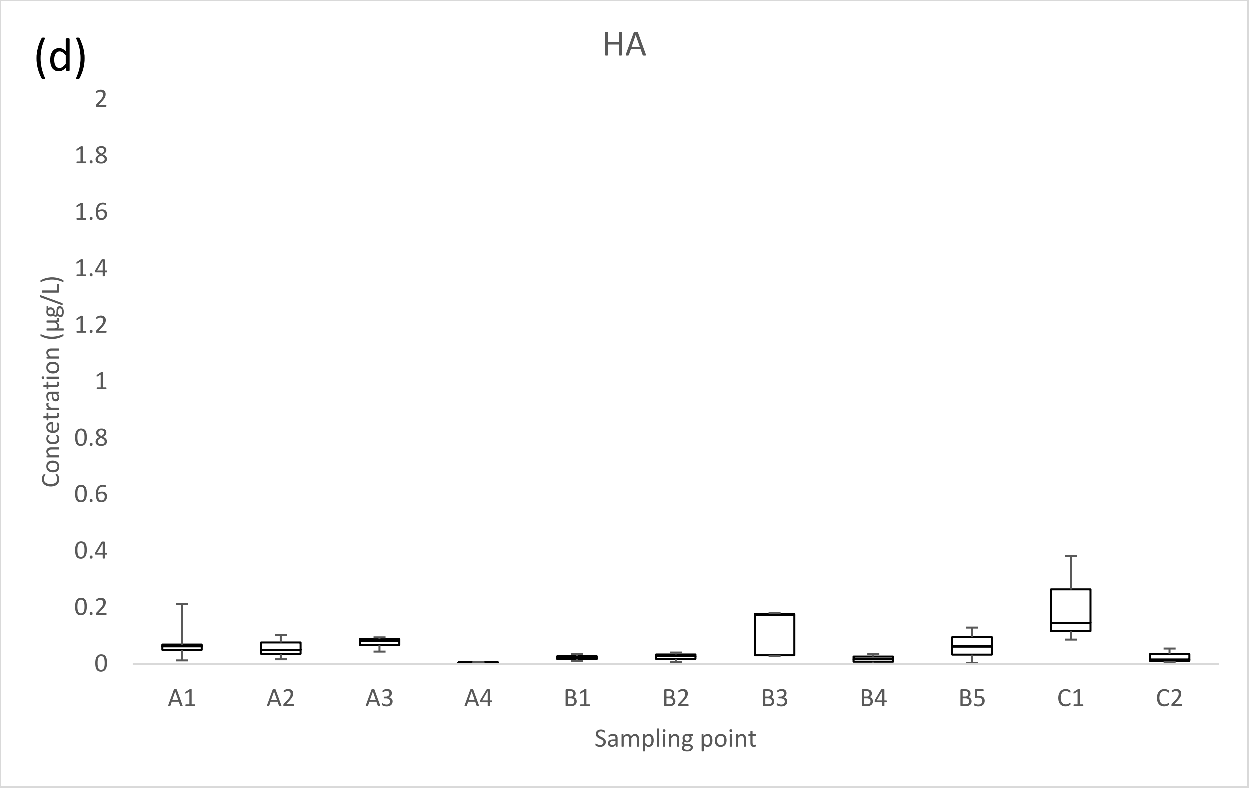
(d)
HA
Concetration (µg/L)
2
1.8
1.6
1.4
1.2
1
0.8
0.6
0.4
0.2
0
A1
A2
A3
A4
B1
B2
B3
B4
B5
C1
C2
Sampling point

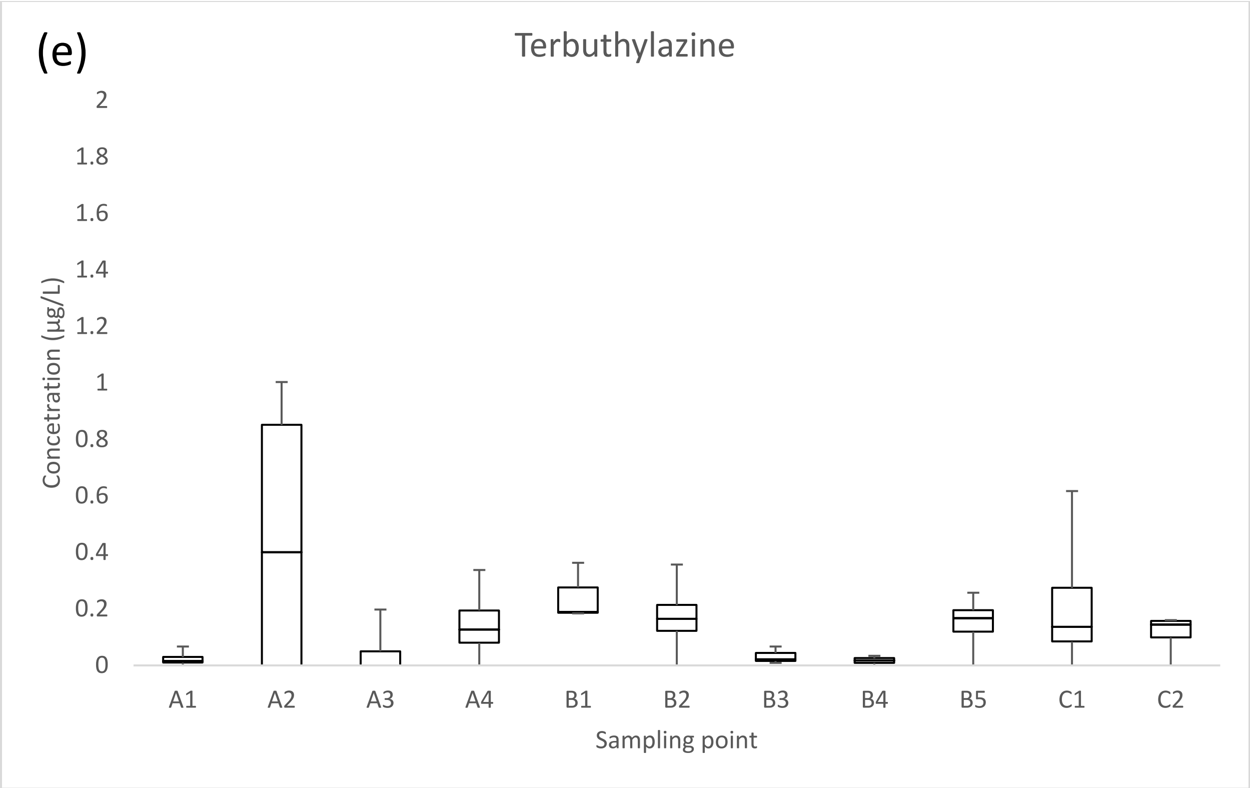

(e)
Terbuthylazine
2
1.8
1.6
1.4
1.2
1
0.8
0.6
0.4
0.2
0
Concetration (µg/L)
A1
A2
A3
A4
B1
B2
B3
B4
B5
C1
C2
Sampling point

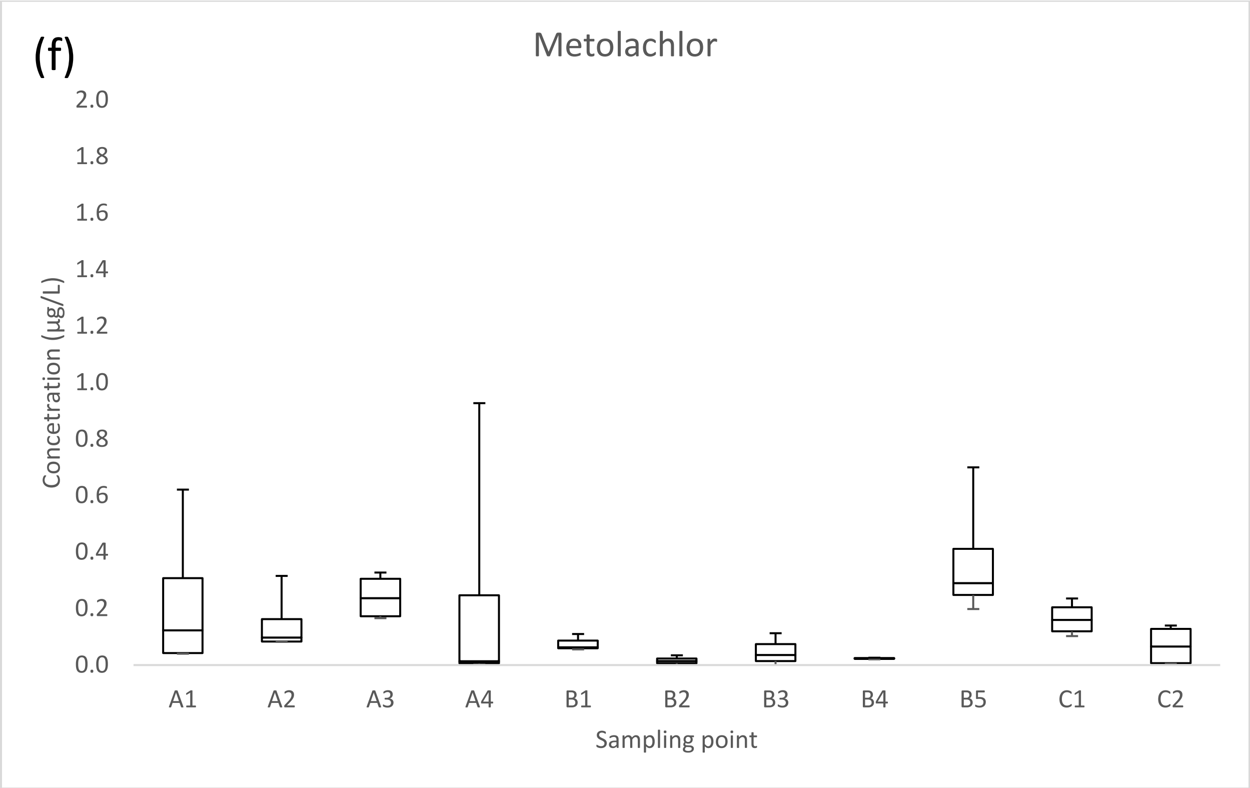

(f)
Metolachlor
2.0
1.8
1.6
1.4
1.2
1.0
0.8
0.6
0.4
0.2
0.0
Concetration (µg/L)
A1
A2
A3
A4
B1
B2
B3
B4
B5
C1
C2
Sampling point

Figure S1. Box-whisker plots of concetrations of pesticides in sampling points: (**a**) atrazine, (**b**) DIA, (**c**) DEA, (**d**) HA, (**e**) terbuthylazine and (**f**) metolachlor. The caps at the end of each box indicate the extreme values (minimum and maximum), the box is defined by the lower and upper quartiles, and the line inside the box denotes the median value. The pesticide concentrations below the LOQs are considered as zero.
